# Separation and Characterization of Antioxidative and Angiotensin Converting Enzyme Inhibitory Peptide from Jellyfish Gonad Hydrolysate

**DOI:** 10.3390/molecules23010094

**Published:** 2018-01-05

**Authors:** Qin Zhang, Chengcheng Song, Jun Zhao, Xiaomei Shi, Meiling Sun, Jing Liu, Yinghuan Fu, Wengang Jin, Beiwei Zhu

**Affiliations:** 1 School of Food Science and Technology, Dalian Polytechnic University, Dalian 116034, China; zhangqin3595@163.com (Q.Z.); 18242054628@163.com (C.S.); shaerxiao@126.com (X.S.); Kexinxiaojia@163.com (M.S.); g815512123@163.com (J.L.); 2 National Engineering Research Center of Seafood, Dalian 116034, China; kittyufo@163.com; 3 School of Light Industry and Chemical Engineering, Dalian Polytechnic University, Dalian 116034, China; 4 Bio-resources Key Laboratory of Shanxi Province, School of Biological Science and Engineering, Shanxi University of Science & Technology, Hanzhong 723001, China; jinwengang@nwafu.edu.cn

**Keywords:** jellyfish gonad protein hydrolysate, peptides, ACE inhibitory activity, antioxidant activity

## Abstract

The gonad of jellyfish (*Rhopilema* *esculentum* *Kishinouye*), containing high protein content with a rich amino acid composition, is suitable for the preparation of bioactive peptides. Jellyfish gonad was hydrolysed with neutral protease to obtain jellyfish gonad protein hydrolysate (JGPH), which was then purified sequentially by ultrafiltration, gel filtration chromatography, and RP-HPLC. The peptides were characterized with HPLC-MS/MS. One peptide with amino acid sequence Ser-Tyr (SY) was identified and synthesized, which showed good ACE inhibitory and antioxidant activity. The IC_50_ of this peptide on DPPH, ·OH, super oxygen anion scavenging activities, and ACE inhibitory activity are 84.623 μM, 1177.632 μM, 456.663 μM, and 1164.179 μM, respectively. The anchor in the binding site of SY and ACE C-domain (ACE-C) was obtained by molecular simulations. The results showed that the dipeptide purified from jellyfish gonad protein hydrolysates can be used as functional food material and is helpful in the study of antioxidant and inhibition of ACE.

## 1. Introduction

In recent years, hypertension has become one of the most common cardiovascular diseases. Substances with antihypertensive activity have gained increasing interest of scientists in various countries. Angiotensin converting enzyme (EC, 3.4.15.1 ACE) is a key enzyme in the regulation of blood pressure. In the renin angiotensin system (RAS), ACE can catalyse angiotensin I to produce angiotensin II, which is a vasoconstrictor with rising-effect [1]. In the kallikrein kinin system (KKS), ACE could degrade bradykinin to an inactive fragment [2]. Therefore, the inhibitors of ACE are concerned with the development of antihypertensive drugs.

Despite the availability of antihypertensive drugs on the market, such as captopril and enazepril, etc. Clinical data indicates that some patients taking synthetic ACE inhibitor have the side-effects of cough and angioedema [3]. Seeking of ACE inhibitory peptides from food protein and using those to replace chemical synthetic inhibitors has become the development trend in this research field [4].

Reactive oxygen species (ROS) are an inevitable product of the biological oxidative metabolism, including all types of unstable free radicals, such as superoxide radicals, hydroxyl radicals, peroxylradicals, and alkoxy radicals, the mediated oxidation reaction will adversely affect cells and the oxidation of intracellular biological macromolecules will lead to cell damage [5]. As a result, free radicals lead to many diseases and aging, such as arthritis, diabetes, and neurodegenerative diseases. Many studies have shown that an excess of ROS would cause and aggravate high blood pressure [6]. Therefore food-derived bioactive molecules with ACE inhibitory and antioxidant activities have the advantages of low cost and no side effects, and are expected to become the ideal functional foods [7].

Recent studies have shown that the enzymatic hydrolysates of different terrestrial proteins usually have antioxidant or ACE inhibitory activities, such as protein from egg white [8], soybean [9], lupin [10], walnut [11], etc. Researchers also prepared hydrolysates from marine protein like *Acaudina molpadioidea* [12], mussel [13], salmon skin [14], squid skin [15], shrimp [16], and jellyfish [17]. A number of peptides were purified from these hydrolysates and the amino acid sequences were identified. The structure-activity relationship of ACE inhibitor peptide was reported in the literature, with most ACE inhibitor peptides containing 2–20 amino acids [18]. 

Gonad is a byproduct of jellyfish processing. Nowadays, the gonad of jellyfish is normally processed as flavourful dishes due. However, the gonad of jellyfish contains approximately 50% protein, which is suitable for the preparation of bioactive peptides. In this paper, we investigate antioxidant and ACE inhibitory activities of jellyfish (*Rhopilema esculentum Kishinouye*) gonad hydrolysates (JGPH). The dipeptide of Ser-Tyr (SY) was purified from JGPH and its antioxidant and ACE inhibitory activity were validated. The inhibition type of this dipeptide and the binding of SY to ACE-C were further investigated.

## 2. Results and Discussion

### 2.1. Amino Acid Composition, ACE Inhibitory Activity and Antioxidative Activity of JGPH

The amino acid composition in hydrolysate is very important for the nutritional value and its effect on functional properties [19]. The freeze-dried JGPH (-P1, -P2, -P3) with different molecular weight grades have been analysed for their composition and content of amino acids. Analysis results are shown in Table 1. From the results, it can be seen that JGPH-P1 and JGPH-P2 obtained by ultrafiltration have similar amino acid composition, and methionine was not detected in JGPH-P3. The slight differences in amino acid composition between the three components may be due to the difference in molecular weight. The total amount of amino acids in the three components was relatively close, accounting for 51.93 ± 2.02%, 52.97 ± 2.72%, and 47.40 ± 1.75%, respectively. Three components have a high percentage of essential amino acids. However, hydrophobic amino acids have significant free radical scavenging activity [20]. Aromatic amino acids and hydrophobic amino acids of peptides play a key role on ACE inhibitory activity [21]. In JGPH-P3, the aromatic amino acids and hydrophobic amino acids were higher than the other two components, accounting for 8.08% and 17.22% of the total amino acids, respectively. Therefore, JGPH-P3 should have good biological activity. Meanwhile, experimental results have shown that the components with molecular weight of less than 1 kDa had higher antioxidant and ACE inhibitory activity (Table 2), which is consistent with the literature reports [21]. Based on the amino acid composition and the results obtained, JGPH-P3 was chosen as the material for separating bioactive peptides with ACE inhibitory activity and antioxidant activity.

### 2.2. Purification of ACE Inhibitory and Antioxidative Peptide

**Figure 1:**
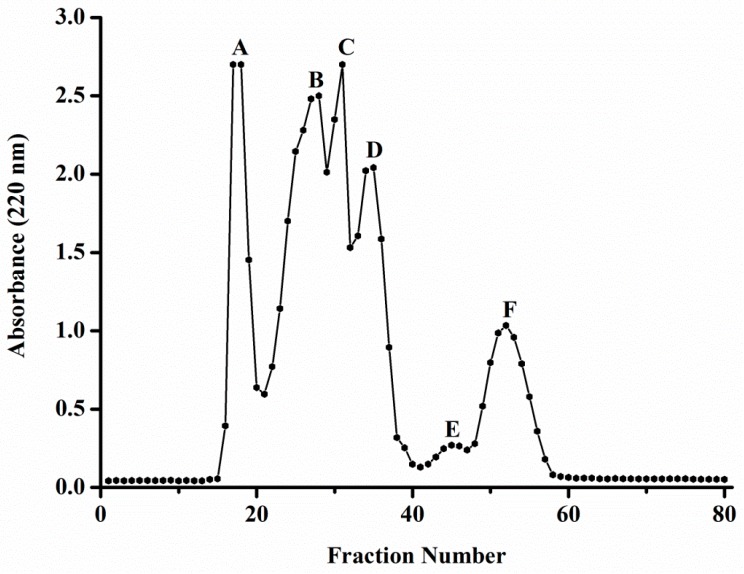
Elution profile of JGPH-P3 using a Sephadex G-25 column. A–F were six major parts by the Sephadex G-25 gel column.

Taking into account the ACE inhibitory and antioxidant activity effect, JPGH-P3 was used for purification and identification of the ACE inhibitory and antioxidant peptide. JPGH-P3 was fractionated by a Sephadex G-25 gel filtration column. As shown in Figure 1, JGPH-P3 was separated into six major parts by the Sephadex G-25 gel column: JGPH-P3A, JGPH-P3B, JGPH-P3C, JGPH-P3D, JGPH-P3E, and JGPH-P3F. The ACE inhibitory activity and DPPH scavenging activity of the obtained six fractions were determined. 

**Figure 2:**
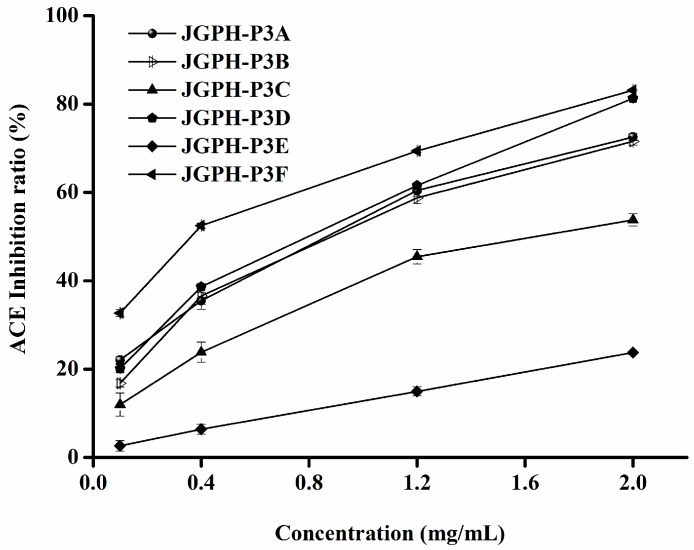
ACE inhibitory activities of JGPH-P3 A–F.

**Figure 3:**
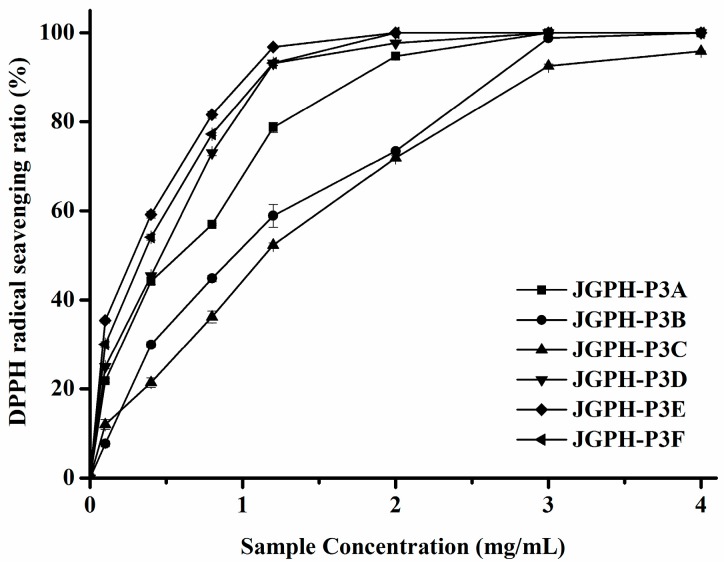
DPPH radical scavenging activity of JGPH-P3 A–F.

ACE inhibitory activity of the fractions, JGPH-P3A to P3F, was determined by the HPLC method and the results are shown in Figure 2. It can be seen from Figure 2 that all of the six fractions show ACE inhibitory activity. Among these samples JGPH-P3F exhibits the highest ACE inhibition activity, the IC_50_ of which was 0.54 mg·mL^−1^, and JGPH-P3D possesses the second highest activity, the IC_50_ for which was 0.96 mg·mL^−1^. The activity of JGPH-P3E was the lowest (Table 3). ESR (electron spin resonance spectroscopy) was used to determine the DPPH scavenging activity of JGPH-P3A, JGPH-P3B, JGPH-P3C, JGPH-P3D, JGPH-P3E, and JGPH-P3F, the results of which are shown in Figure 3. It can be seen from Figure 3 that all of the six parts of JGPH have antioxidant activity. Among these fractions, JGPH-P3E exhibits the maximum scavenging rate and has an IC_50_ of 0.29 mg·mL^−1^, followed by JGPH-P3F and JGPH-P3D, having IC_50_ of 0.38 mg·mL^−1^ and 0.48 mg·mL^−1^, respectively. DPPH scavenging activity of JGPH-P3C was the lowest (Table 3). 

JGPH-P3E had the highest DPPH scavenging activity, but ACE inhibitory activity of it was much lower. JGPH-P3F and JGPH-P3D were selected for further purification because these components have higher ACE inhibitory activity and antioxidant activity.

JGPH-P3 was further purified using RP-HPLC on a C_18_ column with a linear gradient of 0–30% acetonitrile at a flow rate of 1.5 mL·min^−1^. After consecutive chromatography separation and collection, we obtained several samples which were analysed using Q-TOF. The analysis results of samples were dissatisfying, only a fraction of P3D5 had a strong enough response to be detected.

### 2.3. Identification Sequence of the Purified Peptide 

**Figure 4:**
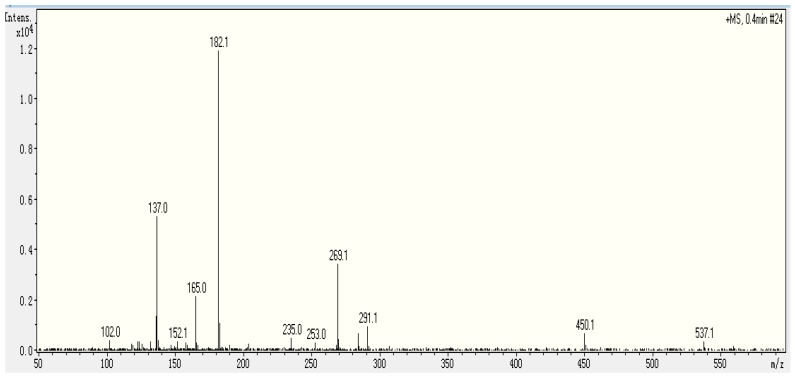
ESI-MS/MS chromatography of P3D5.

The molecular weight of P3D5 was 268.1 Da, which was determined by ESI-MS. To elucidate the structure of this sample, ESI-MS/MS was conducted. The MS spectra of P3D5 showed in Figure 4 indicates that it may be a dipeptide. From the data in Figure 4, the mass to charge ratio of 269.1 can be attributed to Ser-Tyr [M + H], and the mass to charge ratios of 182.1, 165.0, and 137.0 can be assigned to the y1 ion, z1 ion, and tyrosine immonium ion, respectively. Peptide sequencing was performed via manual calculation. This peptide had been reported as an ACE inhibitory purified peptide from *Allium stadium* L. (*garlic*) [22]. As an inhibitory peptide, it was also derived from fermented soybean seasoning [23], porcine skeletal muscle proteins [24], vegetable foods [25], etc. The peptide was also reported as an inhibitor of other enzymes: peptide α-*N*-acetyltransferase [26] and dipeptidyl peptidase IV [27]. The SY isolated from the gonad of jellyfish has two effects on antioxidant and ACE inhibition.

### 2.4. Analysis of Radical Scavenging Activity of Ser-Tyr

**Figure 5:**
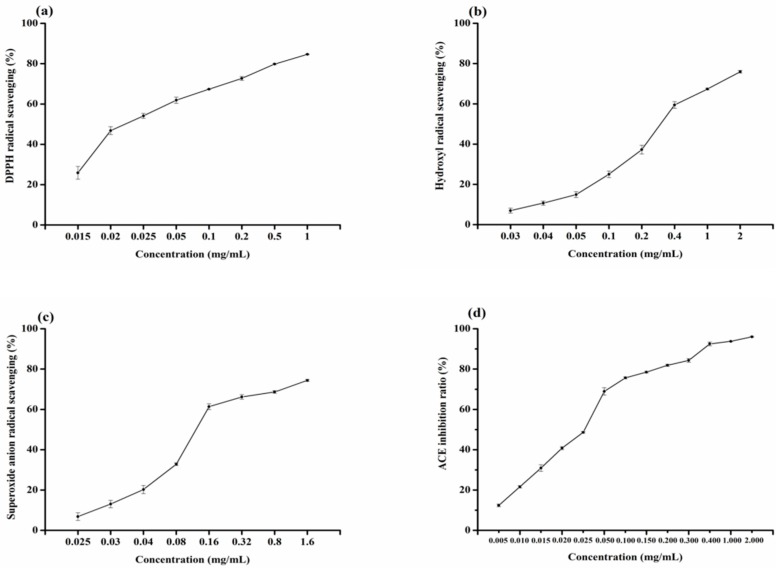
(**a**) DPPH radical scavenging activity of Ser-Tyr. (**b**) Hydroxyl radical scavenging activity of Ser-Tyr. (**c**) Superoxide anion radical scavenging activity of Ser-Tyr. (**d**) ACE inhibitory activity of Ser-Tyr.

To evaluate the antioxidant activities of SY, DPPH, hydroxyl free radical, and superoxide anion scavenging rate of synthetic peptides Ser-Tyr were determined by using electron spin resonance. The results are shown in Figure 5a–c. It can be seen that SY has good antioxidative activity. DPPH radical scavenging activity was the highest, second was superoxide radical scavenging ability, and the hydroxyl radical scavenging ability is relatively weak. Table 4 shows the IC_50_ values of SY for DPPH, hydroxyl, and superoxide radicals. Antioxidant peptides have been reported, such as Val-Lys-Ala-Gly-Phe-Ala-Trp-Thr-Ala-Asn-Gln-Gln-Leu-Ser isolated from tuna backbone protein [28], Leu-Leu-Gly-Pro-Gly-Leu-Thr-Asn-His-Ala and Asp-Leu-Gly-Leu-Gly-Leu-Pro-Gly-Ala-His isolated from marine rotifer [29], Ile-Lys-Lys, Phe-Lys-Lys, and Phe-Ile-Lys-Lys separated from the protease digest of prawn (*Penaeus japonicus*) muscle [30], etc. The study of SY as an antioxidant peptide has not been reported. Compared with the antioxidative peptide Glu-Ser-Thr-Val-Pro-Glu-Arg-Thr-His-Pro-Ala-Cys-Pro-Asp-Phe-Asn isolated from hoki frame protein, with scavenging ability to DPPH, hydroxyl, and superoxide radicals, and IC_50_ values were 41.37 μM, 17.77 μM, and 172.10 μM [31], SY has good scavenging ability to DPPH, hydroxyl and superoxide radicals, and IC_50_ values were 84.623 μM, 1177.632 μM, and 456.663 μM, respectively. SY exhibited high antioxidant activity, possibly due to the hydrophobic amino acids contained in the molecular structure. As reported in the literature, hydrophobic amino acid in peptides can obviously improve the oxidation resistance [32]. SY on the DPPH scavenging radical ability is higher than the other two scavenging radicals, and the scavenging hydroxyl radical may be the most effective defence for the body to fight the disease [31]. The results showed that SY may contribute to antioxidation.

### 2.5. Analysis of ACE Inhibitory Activity of Ser-Tyr

The ACE inhibitory activity of Ser-Tyr was determined. The results are shown in Figure 5d. Compare with the ACE inhibitory peptides that have been reported, dipeptide Gly-Pro isolated from Alaska Pollock skin (IC_50_: 252.63 μM) [33], Tyr-Pro (IC_50_: 720.00 μM) isolated from haemoglobin [34], and Ile-Val-Thr-Asn-Trp-Asp-Asp-Met-Glu-Lys (IC_50_: 2080.00 μM) and Val-Gly-Pro-Ala-Gly-Arg-Pro-Gly (IC_50_: 4660.00 μM) separated from *Voluthar paampullacea perryi* [35]. It can be seen that the SY had a good ACE inhibitory activity; the IC_50_ of which was 1164.179 μM (Table 4). The IC_50_ value may be related to the determined conditions and methods. At low concentration, ACE inhibitory activity of SY showed a positive correlation with the concentration. The ACE inhibitory activity of peptide is closely related to the composition and sequence of amino acids [36]. According to the literature, peptides with high ACE inhibitory activity often possess aromatic amino acids and N-terminal hydrophobic amino acid [37]. The amino terminal of SY is the hydrophobic amino acid tyrosine, indicating that SY may be helpful to inhibit the activity of ACE.

### 2.6. The ACE Inhibition Pattern of Ser-Tyr

**Figure 6:**
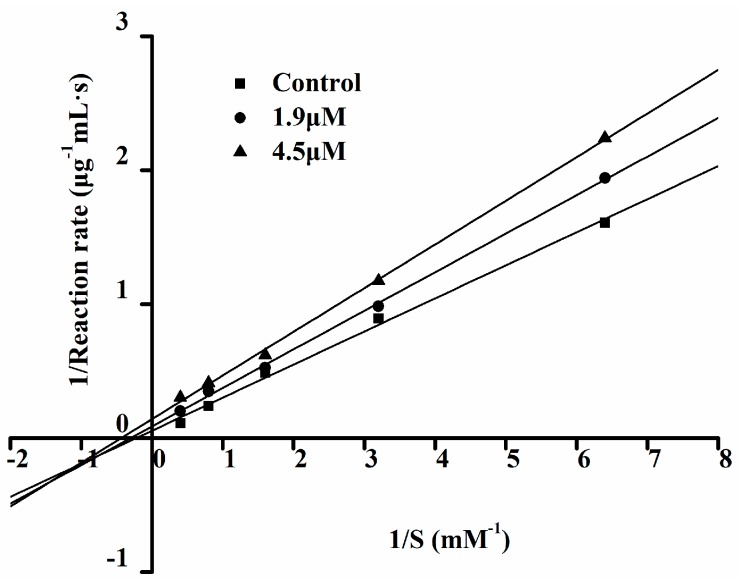
Lineweaver–Burk plots on ACE inhibitory activity of Ser-Tyr.

The Lineweaver–Burk double reciprocal graph method is often used to determine the type of inhibition of an inhibitor [9]. Non-competitive inhibition is that inhibitor which binds with a non-active site of enzyme and that does not have competition with the substrate. Anti-competitive reversible inhibition refers to a complex inhibitor that only binds with an enzyme-substrate compound, thereby inhibiting the enzyme activity. The inhibitory pattern of Ser-Tyr (SY) was identified through Lineweaver–Burk plots (Figure 6), the three straight lines intersected at one point on the 1/S axis, the results showed SY is a non-competitive inhibitor of ACE [38]. This means that the peptide can combine with ACE molecule to produce a dead-end complex, regardless of abundant substrate molecules or not. 

### 2.7. Molecular Docking 

**Figure 7:**
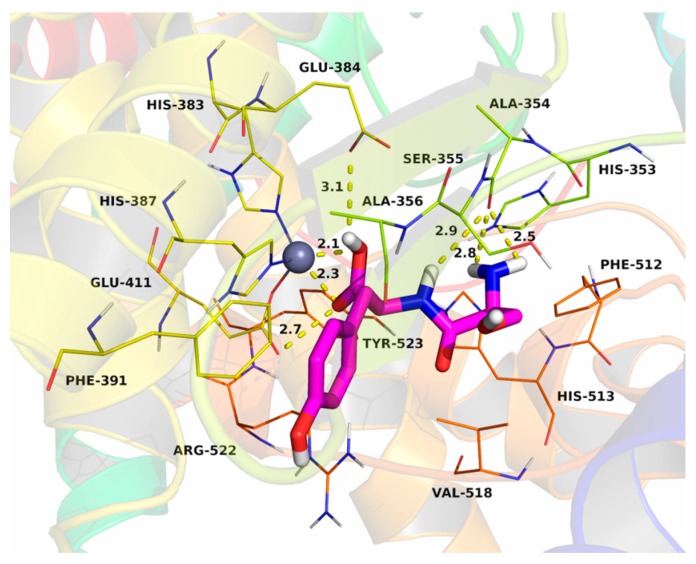
The docking of Ser-Tyr binding with the human ACE-C.

The dipeptide SY was docked into the binding site of the ACE-C and the theoretical binding mode is illustrated in Figure 7. The SY adopted a compact conformation to bind inside of the site of ACE-C. The phenyl group of SY stretched into the hydrophobic pocket that consisted of Phe-391 and Ala-365, forming a stable hydrophobic binding. Detailed analysis showed that the phenyl group of SY formed the CH-π and cation-π interactions with the residue Phe-391 and Arg-522, respectively. The carboxyl group of SY formed two hydrogen bonds with the zinc ion which, in turn, interacted with the residues His-383, His-387, and Glu-411. Additionally, the carboxyl group of SY formed another two hydrogen bonds with the residues Glu-384 and Glu-411. In addition, amide and amino groups of the SY formed three hydrogen bonds with the residue Ala-354. The estimated binding energies were −7.9 kcal·mol^−1^ for the ACE-C. All these interactions helped SY to anchor in the binding site of the ACE-C. The above molecular simulations give us a rational explanation of the interactions between the SY and the human ACE-C, which provided valuable information for further development of the mechanism of action of the SY for inhibiting human ACE.

## 3. Materials and Methods

### 3.1. Chemicals

Jellyfish (*Rhopilema esculentum Kishinouye*) gonads were supplied by Rongfa ShenZhe Co. (Yingkou, China), Neutrase used was purchased from Guangxi Pangbo Co. (Guangxi, China). Angiotensin converting enzyme (from rabbit lung), ACE substrate hippuryl-histidyl-leucine (HHL), hippuric acid (Hip), trifluoroacetic acid (TFA), 1,1-diphenyl-2-picrylhydrazyl (DPPH), 5,5-dimethyl-1-pyrroline *N*-oxide (DMPO), hydroperoxide (HPX), diethylenetriaminepentaacetic acid (DTPA), xanthine oxidase (XOD), and ethylenediaminetetraacetic acid disodium salt (EDTA-Na_2_) were purchased from Sigma (St. Louis, MO, USA). Acetonitrile was purchased from Spectrum chemical reagents Co. (Plainfield, IL, USA). All the other chemicals and solvents used in this study were of the highest analytical grade.

### 3.2. Preparation of Enzymatic Hydrolysates

Neutral protease was employed for the preparation of jellyfish gonad protein hydrolysate under the following conditions: The jellyfish gonad powder was dispersed in deionized water at ratio of 1:10 (*w*/*v*). Neutrase was added to the suspension with a substrate to enzyme dosage of 2500 U/g jellyfish gonads. The hydrolysis was conducted at pH 7.0 through continuous addition of 0.1 M NaOH. After incubation at 45 °C for 2 h, the mixture was heated at 100 °C for 10 min to terminate the enzymatic hydrolysis. After immediately cooling and centrifugation (10,000 rpm/min for 20 min), the supernatant was lyophilized and named JGPH. The obtained lyophilized powder of jellyfish gonad protein hydrolysate (JGPH) was ultrafiltrated through 1 and 3 kDa molecular weight cut-off (MWCO) membranes. The filtrates in each filtration were collected and named JGPH-P1 (more than 3 kDa), JGPH-P2 (1~3 kDa), and JGPH-P3 (less than 1 kDa). Their ACE inhibitory activities and radical scavenging activity were measured.

### 3.3. Determination of Amino Acid Composition

A sample was hydrolysed with 3 mL of 6 M HCl at 110 °C for 24 h. The hydrolysed sample was evaporated on a water bath, then diluted to 25 mL with derivatization buffer. Before analysis, the solution was filtered through a 0.45 μm membrane filter. The treated sample was derived using an Elite-AAk Reagent Kit (according to Elite-AAk chemistry package instruction manual). HPLC analysis was performed with the Elite-AAK (Dalian elite analytical instruments Co., Ltd., DaLian, China). The separation module was equipped with an Elite 1201 UV detector and amino acids were separated on an Elite-AAK amino acid analytical column (ODS C_18_, 200 mm × 4.6 mm). According to the peak area in comparison with the standard, amino acid contents were calculated.

### 3.4. Purification of Jellyfish Gonad Peptides

The activity of ACE inhibitory peptides and antioxidant peptides with high activity were closely related to the molecular weight, peptides with small molecular weight are usually highly active [39]. Among the components obtained by Sephadex G-25 separation, JPGH-P3, showing the highest ACE inhibitory activity and antioxidant activity, was selected as the separation and purification material. JGPH-P3 freeze-dried powder (50 mg) was dissolved in 1 mL water. This sample solution was loaded onto pre-equilibrated Sephadex G-25 gel (1.6 cm × 58 cm) chromatography at a flow rate of 0.29 mL·min^−1^ with deionized water as the elution reagent. Elution curves were obtained by measuring the absorbance at 220 nm using a UV spectrophotometer. The fraction that exhibited same absorbance was collected and then lyophilized. The obtained fractions were named JGPH-P3A, JGPH-P3B, JGPH-P3C, JGPH-P3D, JGPH-P3E, and JGPH-P3F.

The sample JGPH P3D was further purified using reverse-phase HPLC (RP-HPLC) (Elite). RP-HPLC was carried out using a C_18_ semi-preparative column (particle size of 5 μm, 10 mm × 250 mm, Tigerkin, Beijing Greenherbs Science and Technology Development Co., Ltd., Beijing, China) with a gradient elution. Sample volumes of 20 μL were injected into C_18_ and eluted in 30 min using a gradient with 100% A (0.05% TFA in water) for 5 min followed by a liner increase to 30% B (acetonitrile) over the next 25 min. The flow rate was adjusted to 1.5 mL/min. The UV absorbance of the eluent was monitored at 220 nm and eluted fractions were collected. The main component collected was named P3D5. The chromatographic column was conditioned with 100% of eluent A, after which 15 μL of the sample was injected into the C_18_ column and eluted with eluent A for 6 min and with increasing eluent B concentration as follows: 0% (*v*/*v*) from 0–6 min to 0–100% (*v*/*v*) from 6 to 14 min.

### 3.5. DPPH Radical Scavenging Activity

The DPPH radical scavenging activity was measured according to the method described by Chen Yong et al. [40]. Two-hundred micromoles of DPPH, 95% ethanol solution, and different concentrations of samples were reacted in the dark for 30 min. The reactant was immediately inhaled by capillaries into the resonant cavity and scanning detection was conducted. Glutathione (GSH) was used as a positive control. Scanning conditions were as follows: central magnetic field strength, 3418.78 G; microwave power, 6.23 mW; microwave frequency, 9.44 GHz; magnification, 1.0 × 10^5^; amplitude modulation, 1.0 G; modulation frequency of 100 kHz; time constant, 163.84 ms; and transformation time, 80 ms. DPPH radical scavenging activity (DRSA) was calculated based on the following Equation (1), in which *h_s_* and *h*_0_ were the height of the third resonance peak in the presence and absence of the sample, respectively:

(1) $$\mathrm{DRSA}(\%)=\frac{h_{0}-h_{s}}{h_{0}}\times 100$$

The IC_50_ value was defined as the concentration of peptides that could scavenge 50% of the DPPH radical activity.

### 3.6. Hydroxyl Radical Scavenging Activity

Hydroxyl radicals were generated by iron-catalysed Haber-Weiss reaction (Fenton-driven Haber-Weiss reaction) and the generated hydroxyl radicals rapidly reacted with a nitrone spin trap DMPO (dimethyl pyridine *N*-oxide) [41]. The resultant DMPO-OH adduct was detectable by an ESR spectrometer. Peptide (40 μL) with various concentrations were mixed with DMPO (1 M, 5 μL), EDTA Na_2_-Fe^2+^ (6 mM, 10 μL) and H_2_O_2_ (6%, 8 μL) in a phosphate buffer solution (0.15 M, pH 7.4), and then transferred into a 100 μL quartz capillary tube. After 2.5 min, the spectrum was recorded using an ESR spectrometer. Deionized water was used instead of the sample as a control. GSH was used as a positive control. Experimental conditions were as follows: magnetic field, 3368.38 G; microwave power, 7.25 mW; microwave frequency 9.44 GHz; magnification, 1.0 × 10^5^; amplitude modulation, 1.0 G; modulation frequency, 100 kHz; time constant, 81.92 ms; and sweep time, 40 ms. Hydroxyl radical scavenging activity (HRSA) was calculated based on the following Equation (2), in which *h*_0_ and *h_s_* were the height of the second resonance peak in the presence and absence of the sample, respectively:

(2) $$\mathrm{HRSA}(\%)=\frac{h_{0}-h_{s}}{h_{0}}\times 100$$

The IC_50_ value was defined as the concentration of peptides that could scavenge 50% of the hydroxyl radical.

### 3.7. Superoxide Radical Scavenging Activity

The hypoxanthine xanthine oxidase (HPX-XOD) reaction system based on $O_{2}^{-}$ peptide (31.8 μL) with various concentrations was mixed with DMPO (1 M, 5 μL), HPX (5 mM), DTPA(10 mM, 5 μL), and XOD (1 U·mL^−1^, 1 μL) in a phosphate buffer solution (0.05 M, pH 7.4), and then transferred into a 100 μL quartz capillary tube. Deionized water was used instead of the sample as a negative control. Experimental conditions were as follows: magnetic field, 3367.95 G; microwave power, 1.16 mW; microwave frequency, 9.44 GHz; magnification, 1.0 × 10^5^, amplitude modulation, 1.0 G; modulation frequency, 100 kHz; time constant, 2521.44 ms; and sweep time, 480 ms. Superoxide radical scavenging activity (SRSA) was calculated based on the following Equation (3), in which *h*_0_ and *h_s_* are the height of the first resonance peak in the presence and absence of the sample, respectively:

(3) $$\mathrm{SRSA}(\%)=\frac{h_{0}-h_{s}}{h_{0}}\times 100$$

The concentration of peptides needed to scavenge superoxide radical activity by 50% is defined as the IC_50_ value.

### 3.8. Measurement of ACE Inhibition Activity

The ACE inhibitory activity of samples was determined according to the references of Cushman [42] and Liu [43]. The ACE inhibition rate was determined based on the reduced percentage of the peak area of hippuric acid (HA), using an adapted HPLC method [15] with slight modifications. A sample solution (20 µL) was added to 50 µL of substrate HHL (5 mM Hip-His-Leu in 0.1 M borate buffer containing 0.3 M NaCl at pH 8.3), the mixture was pre-incubated at 37 °C for 5 min. The reaction was initiated by adding 20 µL of ACE solution (0.1 U·mL^−^^1^ in 0.1 M borate buffer containing 0.3 M NaCl at pH 8.3) and incubated for 60 min at the same temperature. Ten microliters of HCl (0.2 M) was added to terminate the reaction. The control test was conducted using 20 μL of deionized water instead of the sample. Ten microliters of the reaction solution were injected directly into a Ghall 12S05-2546 C_18_ column (250 mm × 4.6 mm). The mobile phase was 25% acetonitrile and 75% water with 0.05% trifluoroacetic acid (TFA). The flow rate was 0.5 mL·min^−1^ and the spectra was collected at 228 nm to evaluate the degree of inhibition of ACE activity. The inhibition activity was calculated using the following Equation (4):

(4) $$\mathrm{ACE}\;\mathrm{inhibition}\;\left(\%\right)=\frac{A_{s}{\text{−}A}_{c}}{A_{s}}\text{×}100\;$$

where *A_s_* is the area of Hip after inhibitor added, and *A_c_* is the area of Hip in the control test.

The IC_50_ value was defined as the concentration of inhibitor that could inhibit 50% of the ACE activity.

### 3.9. Characterization of Peptides

The molecular weight and amino acid sequence of the target peptides were determined by using a quadrupole time-of-flight (Q-TOF) mass spectrometer (Micromass Co., Manchester, UK). The sample was operated in the positive electrospray ionization mode via the electrospray interface. The drying temperature (200 °C) and ESI nebulizing gas (44 psi) used was high-purity nitrogen. Spectra were recorded over the mass/charge (*m*/*z*) range of 50–2000. About three spectra were averaged in the MS and MS/MS analyses. 

### 3.10. Synthesis and Purity Identification of SY

In order to evaluate the ACE inhibitory activity of the purified peptide (SY), the peptide with the same sequence was synthesized by solid-phase synthesis. The peptide synthesis was commissioned by the Shanghai Gil Biochemical Co. Ltd. (Shanghai, China) with purity of 98% and its ACE inhibitory activity was determined.

### 3.11. Determination of the Inhibition Mode on ACE

Basic conditions of the experiment were the same as the assay of ACE inhibitory activity. ACE inhibitory activity was measured under different concentrations of the substrate HHL (0.015625 mM, 0.3125 mM, 0.625 mM, 1.25 mM, and 2.5 mM). The kinetics of ACE in the presence of the inhibitor was determined by Lineweaver–Burk plots [38]. 

### 3.12. Molecular Docking

Molecular docking study was performed to investigate the binding mode between the dipeptide SY and the human angiotensin converting enzyme (ACE) using AutodockVina 1.1.2 (Scripps Research Institute, La Jolla, CA, USA) [44]. The crystal structures of human ACE C-domain (ACE-C, PDBID: 2OC2) was derived from the RCSB Protein Data Bank [45]. The 3D structure of SY was drawn by Chem Bio Draw Ultra 12.0 and ChemBio3D Ultra 12.0 software packages (Cambridge Soft, Cambridge, MA, USA). The AutoDock Tools 1.5.6 package [46,47] was employed to generate the docking input files. The search grid of ACE-C was identified as center_x: 41.678, center_y: 38.089, and center_z: 46.648 with dimensions size_x: 15, size_y: 15, and size_z: 15. The value of exhaustiveness was set to 20. For Vina docking, the default parameters were used if it was not mentioned. The best-scoring pose judged by the Vina docking score was chosen and visually analysed using PyMoL1.7.6 software (1.3r1, DeLano Scientific LLC, South San Francisco, CA, USA) [48].

### 3.13. Statistical Analysis

All the activity tests were conducted in triplicate. The results were recorded as means ± standard deviation. Differences between the experimental groups were determined by the Student’s *t*-test, and *p*-values less than 0.05 were considered to be significant.

## 4. Conclusions

An antioxidant and ACE inhibitory dipeptide, Ser-Tyr, was successfully purified from neutrase hydrolysate of jellyfish (*Rhopilema esculentum Kishinouye*) gonad protein and characterized using HPLC MS/MS. The dipeptide exhibited DPPH, hydroxyl, and superoxide radical scavenging effects with IC_50_ 84.623 μM, 1177.632 μM, 456.663 μM, respectively. The dipeptide showed ACE inhibitory activity with IC_50_ values 1164.179 μM, and its ACE inhibitory pattern was showed to be a non-competitive inhibition pattern. Molecular simulations give us rational explanations of the interactions between SY and the human ACE-C. It could be suggested that this peptide has the potential to be used in functional food preparations, which target at reducing ACE activity and oxidative-stress-mediated heart diseases.

## Figures and Tables

**Table 1:** Amino acid composition of JGPH (-P1, -P2, -P3).

Types of Amino Acids	JGPH-P1(g/100 g)	JGPH-P2(g/100 g)	JGPH-P3(g/100 g)
Asp	4.43 ± 0.02 ^b^	4.77 ± 0.33 ^b^	3.68 ± 0.06 ^a^
Glu	5.96 ± 0.06 ^b^	6.20 ± 0.20 ^b^	4.51 ± 0.18 ^a^
Ser	2.20 ± 0.07 ^a^	2.18 ± 0.19 ^a^	1.91 ± 0.05 ^a^
Arg	2.79 ± 0.03 ^ab^	3.20 ± 0.26 ^b^	2.66 ± 0.03 ^a^
Gly	4.83 ± 0.23 ^a^	4.50 ± 0.22 ^a^	4.34 ± 0.16 ^a^
Thr ^A^	2.60 ± 0.26 ^a^	2.26 ± 0.26 ^a^	1.91 ± 0.20 ^a^
Pro ^C^	2.06 ± 0.03 ^b^	1.72 ± 0.12 ^a^	1.52 ± 0.03 ^a^
Ala ^C^	2.67 ± 0.09 ^a^	2.95 ± 0.04 ^a^	2.74 ± 0.17 ^a^
Val ^AC^	2.63 ± 0.01 ^b^	2.76 ± 0.07 ^c^	2.44 ± 0.02 ^a^
Met ^AC^	0.85 ± 0.00 ^a^	0.99 ± 0.02 ^b^	1.01 ± 0.03 ^b^
Cys	0.22 ± 0.01	0.03 ± 0.01	—
Ile ^AC^	2.01 ± 0.01 ^a^	2.12 ± 0.27 ^a^	1.99 ± 0.07 ^a^
Leu ^AC^	3.00 ± 0.26 ^a^	3.42 ± 0.06 ^a^	3.45 ± 0.02 ^a^
Trp ^ABC^	3.22 ± 0.16 ^a^	3.48 ± 0.13 ^a^	4.01 ± 0.03 ^b^
Phe ^ABC^	0.06 ± 0.01 ^a^	0.05 ± 0.01 ^a^	0.06 ± 0.01 ^a^
His	4.95 ± 0.58 ^a^	4.39 ± 0.20 ^a^	4.23 ± 0.51 ^a^
Lys ^A^	5.91 ± 0.11 ^b^	6.25 ± 0.04 ^c^	4.92 ± 0.12 ^a^
Tyr ^B^	1.55 ± 0.07 ^a^	1.69 ± 0.29 ^a^	4.01 ± 0.03 ^a^
total	51.93 ± 2.02 ^b^	52.97 ± 2.72 ^b^	47.40 ± 1.75 ^a^

Note: the uppercase letter ^A^ stands for the human body essential amino acids; the uppercase letter ^B^ stands for aromatic amino acids; the uppercase letter ^C^ stands for hydrophobic amino acids. Data are presented as means ± SD (*n* = 3), different letters (^a–c^) indicate statistical different kinds of amino acid content differences among three components (*p* < 0.05).

**Table 2:** DPPH radical scavenging activity and ACE inhibitory activity of JGPH (-P1, -P2, -P3).

Components	DPPH Scavenging Activity (%) ^1^	ACE Inhibition Activity (%) ^2^
JGPH-P1	39.87 ^A^	79.91 ^a^
JGPH-P2	65.68 ^B^	79.98 ^b^
JGPH-P3	87.35 ^C^	87.35 ^c^

Note: ^1^ The concentration of sample was 2.5 mg/mL. ^2^ The concentration of sample was 2.0 mg/mL. Data are presented as means ± SD (*n* = 3), significant difference of DPPH scavenging activity is marked with different uppercase letters (^A–C^) (*p* < 0.05), significant difference of ACE inhibitory activity is marked with different lowercase letters (^a–c^) (*p* < 0.05).

**Table 3:** IC_50_ values of JGPH-P3 A–F.

Fractions	IC_50_ of DPPH Scavenging Activity (mg/mL)	IC_50_ of ACE Inhibitory Activity (mg/mL)
JGPH-P3A	0.62 ± 0.0071 ^D^	1.01 ± 0.0379 ^b^
JGPH-P3B	0.95 ± 0.0314 ^E^	1.07 ± 0.0376 ^b^
JGPH-P3C	1.56 ± 0.0206 ^F^	1.67 ± 0.0412 ^c^
JGPH-P3D	0.48 ± 0.0005 ^C^	0.96 ± 0.0197 ^b^
JGPH-P3E	0.29 ± 0.0084 ^A^	4.38 ± 0.0154 ^d^
JGPH-P3F	0.38 ± 0.0095 ^B^	0.54 ± 0.0241 ^a^

Note: Data are presented as means ± SD (*n* = 3), significant difference of DPPH scavenging activity (IC_50_) is marked with different uppercase letters (^A–F^) (*p* < 0.05), significant difference of ACE inhibitory activity (IC_50_) is marked with different lowercase letters (^a–d^) (*p* < 0.05).

**Table 4:** IC_50_ values of the Ser-Tyr.

Bioactivities	IC_50_ (μM)
ACE inhibitory	1164.179 ± 0.37
DPPH radical	84.623 ± 0.75
GSH (DPPH radical)	162.695 ± 0.32
Hydroxyl radical	1177.632 ± 1.86
GSH (hydroxyl radical)	7306.616 ± 0.32
Superoxide radical	456.663 ± 2.23
